# Optimization of Viable Glioblastoma Cryopreservation for Establishment of Primary Tumor Cell Cultures

**DOI:** 10.1089/bio.2020.0050

**Published:** 2021-02-09

**Authors:** Klara Valyi-Nagy, Fay Betsou, Alexandru Susma, Tibor Valyi-Nagy

**Affiliations:** ^1^ Department of Pathology, University of Illinois at Chicago, Chicago, Illinois, USA.; ^2^ ISBER Biospecimen Science Working Group, Vancouver, British Columbia, Canada.; ^3^ Integrated BioBank of Luxembourg, Dudelange, Luxembourg.

**Keywords:** cryopreservation, glioblastoma, primary culture

## Abstract

***Background:*** Technologies related to the establishment of primary tumor cell cultures from solid tumors, including glioblastoma, are increasingly important to oncology research and practice. However, processing of fresh tumor specimens for establishment of primary cultures on the day of surgical collection is logistically difficult. The feasibility of viable cryopreservation of glioblastoma specimens, allowing for primary culture establishment weeks to months after surgical tumor collection and freezing, was demonstrated by Mullins et al. in 2013, with a success rate of 59% that was not significantly lower than that achieved with fresh tumor tissue. However, research targeting optimization of viable glioblastoma cryopreservation protocols for establishment of primary tumor cultures has been limited.

***Objectives:*** The objective of this study was to optimize glioblastoma cryopreservation methods for viable cryobanking and to determine if two-dimensional (2D) or three-dimensional (3D) culture conditions were more supportive of glioblastoma growth after thawing of frozen tumor specimens.

***Methods:*** Portions of eight human glioblastoma specimens were cryopreserved by four different protocols differing in the time of enzymatic digestion (before or after cryopreservation), and in the type of cryopreservation media (CryoStor CS10 or 10% dimethyl sulfoxide and 90% fetal calf serum). After 1 month, frozen tissues were thawed, enzymatically digested, if not digested before, and used for initiation of 2D or 3D primary tumor cultures to determine viability.

***Results:*** Among the tested cryopreservation and culturing protocols, the most efficient combinations of cryopreservation and culture were those associated with the use of CryoStor CS10 cryopreservation medium, enzymatic digestion before freezing, and 2D culturing after thawing with a successful culture rate of 8 out of 8 cases (100%). Two-dimensional cultures were in general more efficient for the support of tumor cell growth after thawing than 3D cultures.

***Conclusions:*** This study supports development of evidence-based viable glioblastoma cryopreservation methods for use in glioblastoma biobanking and research.

## Introduction

Glioblastoma is the most common malignant primary brain tumor and is the focus of extensive basic and clinical research efforts targeting improved therapies.^1,2^ Technologies related to the establishment of primary low passage tumor cultures are increasingly important to oncology research and practice.^3–7^ However, processing of fresh tumor specimens for the establishment of primary cultures on the day of surgical collection is logistically difficult. Importantly to tissue biorepository practices, viable cryopreservation of glioblastoma specimens allowing for primary culture establishment months to years after surgical tumor collection and freezing has been demonstrated with a success rate not significantly lower than that achieved with fresh tumor tissue.^8^ Specifically, the success rate of primary culture establishment after cryopreservation in that study (59%) was similar to the success rate when using fresh tissue (63%). Furthermore, no relevant molecular or phenotypic differences between cell lines established from fresh or viable frozen tissue were observed.^8^ Following this study, research targeting optimization of viable glioblastoma cryopreservation protocols for establishment of primary tumor cultures has been limited. The aim of our study was to optimize glioblastoma cryopreservation methods for viable cryobanking relative to two critical factors, digestion conditions, and cryopreservation media, in the scope of two-dimensional (2D) or three-dimensional (3D) post-thaw cultures. We explored the use of two different cryopreservation media (CryoStor CS10 medium and 10% dimethyl sulfoxide [DMSO] and 90% fetal calf serum [FCS]), and the timing of tissue digestion, before or after freezing. We assessed the impact of these two critical parameters on the success rate of 2D or 3D post-thaw primary cultures from eight glioblastoma specimens.

## Materials and Methods

### Cryopreservation

**FIG. 1.**
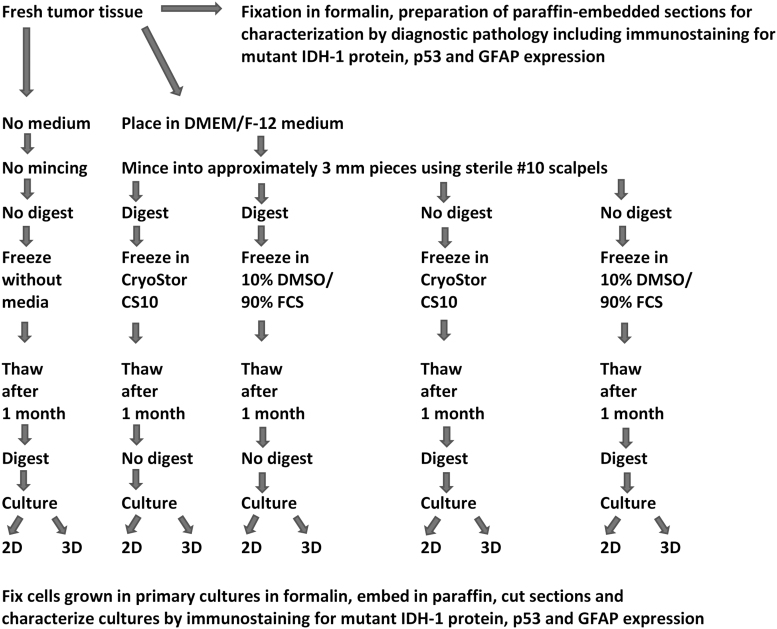
Schematic presentation of tested fresh human glioblastoma cryopreservation protocols and protocols for establishment and characterization of primary glioblastoma cultures after thawing of specimens. 2D, two-dimensional; 3D, three-dimensional; DMSO, dimethyl sulfoxide; FCS, fetal calf serum.

With written patient consent, portions of fresh human glioblastoma specimens that were not needed for pathology diagnosis from eight patients (Table 1) were either snap-frozen in liquid nitrogen without cryopreservation media or were processed for cryopreservation by four cryopreservation protocols with different specifications in the use of enzymatic digestion before or after cryopreservation and in the type of cryopreservation media (Fig. 1). Protocols used were approved by the Institutional Review Board (IRB) of the University of Illinois at Chicago and were in accordance with the Declaration of Helsinki 1975, as revised in 2008. All tissue specimens were first placed in DMEM/F-12 medium (Sigma-Aldrich) with 2 mM glutamine (Gibco, Gaithersburg, MD) and penicillin-streptomycin (Sigma-Aldrich) and minced with the use of sterile #10 scalpels into ∼3 mm pieces within 30 minutes of specimen resection (Fig. 1).

**Table 1.** Clinical Information for Glioblastoma Specimens Studied

Case no.	Patient age (years)	Patient gender	GBM location	IDH status	IDH R132H immunostain	Previous treatment
#1	47	Male	R parietal	Wild type	−	−
#2	46	Male	R frontal	Wild type	−	−
#3	38	Male	R frontal	Mutant	+	−
#4	64	Female	R temporal	Wild type	−	−
#5	35	Female	R frontal	Mutant	+	−
#6	73	Male	R temporal	Wild type	−	−
#7	58	Female	R frontal	Wild type	−	−
#8	57	Male	R frontal	Mutant	+	−

GBM, glioblastoma; IDH, isocitrate dehydrogenase.

For the specimens submitted to enzymatic digestion before freezing, media were then replaced with 2 mL per 0.5 g minced tissue of freshly prepared Enzymatic Tissue Dissociation Media solution (5 mL 0.05% Trypsin/EDTA, 2.5 mL Hank's Balanced Salt Solution [Sigma-Aldrich]—calcium and magnesium free, and 2.5 mL Collagenase IV [ThermoFisher Scientific] stock solution [2000 U/mL in HBBS with calcium and magnesium]). The tissue specimens were digested under rotation for 10 minutes at 37°C in a tissue culture incubator. Digestion was stopped by addition of two volumes of stop solution (5 mL trypsin inhibitor solution, 5 mL DMEM/F12, 2 μL of 5000 U/mL DNase I [Sigma Aldrich; made in HBBS, calcium and magnesium free]) followed by filtering out of undigested material with a 100 μm strainer (BD Biosciences), centrifugation at 800 *g* for 5 minutes at room temperature, and suspension and washing of pellets in DMEM/F-12 medium.

Tissue specimens with or without prefreeze enzymatic digestion were suspended in either CryoStor CS10 (BioLife Solutions) or 10% DMSO (Sigma-Aldrich) and 90% FCS (Fisher, Ontario, Canada) cryopreservation media, frozen in these media with the use of Nalgene Mr. Frosty Cryo 1°C Freezing Containers (Thermo Scientific) at controlled rate cooling, −1°C per minute until reaching −80°C, and then stored long-term in liquid nitrogen vapor phase until further studies (Fig. 1).

### Establishment of primary glioblastoma cultures using cryopreserved tissues

**FIG. 2.**
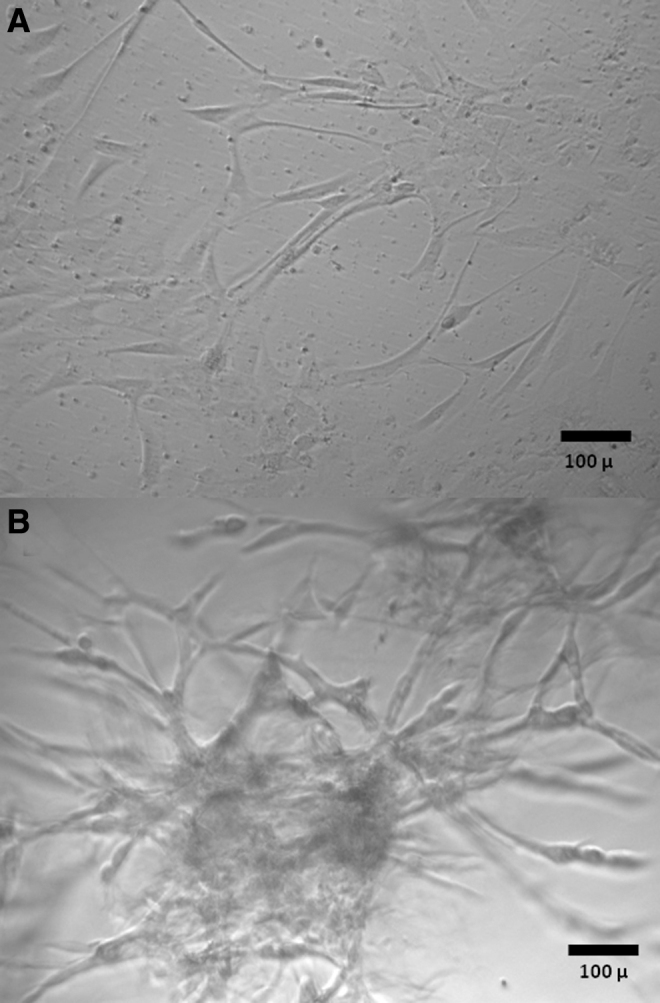
Morphologic features of primary glioblastoma cultures in 2D **(A)** and 3D **(B)** conditions observed and photographed in an inverted microscope. Magnification 200 × .

Snap-frozen glioblastoma samples and glioblastoma samples cryopreserved by the four different cryopreservation methods were thawed at 37°C and were either directly processed for 2D or 3D cultures or were first digested as described in the previous section (Fig. 1). For 2D cultures, samples were placed on 24-well tissue culture plates and cultured in DMEM/F-12 medium (Sigma-Aldrich) with 2 mM glutamine (Gibco) and penicillin-streptomycin (Sigma-Aldrich), supplemented with 10% FCS (Fisher), 20 ng/mL human recombinant epidermal growth factor (EGF; Stem Cell Technologies), 10 ng/mL βFGF (Stem Cell Technologies), and 2 μg/mL Heparin. For 3D cultures, 24-well plates were coated with Matrigel (Corning, Corning, NY) and samples were cultured in DMEM/F-12 medium (Sigma-Aldrich) with 2 mM glutamine (Gibco) and penicillin-streptomycin (Sigma-Aldrich), supplemented with 10% FCS (Fisher), 20 ng/mL human recombinant EGF (Stem Cell Technologies), 10 ng/mL basic fibroblast growth factor (βFGF) (Stem Cell Technologies), and 2 μg/mL Heparin mixed with Matrigel (5% Matrigel/95% medium). Two-dimensional and 3D cultures were observed for cell growth under an inverted microscope. Two-dimensional cultures with cell attachment and growth (Fig. 2A) were trypsinized and transferred to new wells on 24-well tissue culture plates and cultured for additional passages. Three-dimensional cultures demonstrating cell growth (Fig. 2B) were treated with 20 U/well of Dispase (Fisher Scientific) and cells were transferred to new wells on 24-well tissue culture plates and cultured for additional passages in 2D and 3D. The acceptance criteria for successful establishment of primary cultures were samples with cell growth in secondary plates covering at least 50% of the plate. The percentage of cell growth coverage of plates was established by viewing cultures daily under an inverted microscope and recording the average of the estimated coverage of the surface of ten 10 × microscopic fields by cells.

### Characterization of primary cultures derived from cryopreserved samples for GFAP, R132H mutant IDH-1 protein, and p53 expression

To determine if cells in primary cultures derived from cryopreserved samples were expressing GFAP, the R132H mutant form of the IDH-1 protein and p53 in a pattern corresponding to the one documented in the diagnostic pathology report of the original tumor specimen, cells were removed with a sterile cell scraper, fixed in 10% formalin, centrifuged at 1200 rpm, embedded in paraffin, and sectioned. Five micron sections were processed for immunostaining using a Leica Bond Automated Immunostainer to determine the expression of GFAP, the R132H mutant form of the IDH-1 protein, and p53 in the cells.

## Results

### Success rate of establishment of primary glioblastoma cultures following the use of different cryopreservation protocols and 2D or 3D culture conditions

Portions of eight human glioblastoma specimens were either snap-frozen in liquid nitrogen without cryopreservation media or were cryopreserved by four different protocols differing in the use of enzymatic digestion before or after cryopreservation and in the composition of cryopreservation media (CryoStor CS10 or 10% DMSO and 90% FCS) (Fig. 1). After 1 month, frozen tissues were thawed, enzymatically digested if not digested before, and used for initiation of 2D or 3D primary tumor cultures to determine viability (Fig. 1). No primary cultures could be isolated from glioblastoma specimens that had been snap-frozen without the use of cryopreservation media. Among the tested cryopreservation and culturing protocols, the most efficient combination of cryopreservation and culture was that associated with the use of CryoStor CS10 cryopreservation medium, enzymatic digestion before freezing, and 2D culturing after thawing, with a successful culture rate of 8 out of 8 cases (100%) (Table 2 and Fig. 2). The combination of cryopreservation with the use of CryoStor CS10 medium, enzymatic digestion after freezing, and 2D post-thaw culturing showed slightly lower successful culture rate of 7 out of 8 cases (87.5%) (Table 2). Protocols associated with the use of 10% DMSO and 90% FCS cryopreservation medium, enzymatic digestion before or after freezing, and 2D culturing after thawing were associated with a lower successful culture rate of 5 out of 8 cases (62.5%) (Table 2). Two-dimensional cultures were more efficient for the support of tumor cell growth after thawing than 3D cultures (Table 2).

**Table 2.** Success Rates of Establishment of Primary Glioblastoma Cultures Following Different Cryopreservation Protocols and Post-Thawing Culture Methods

Specimen no.	Cryopreservation medium used	Timing of enzymatic digestion	Detection of cell growth (+) or no growth (−) after thawing of frozen tissues and culturing in	
			2D cultures	3D cultures
GBM #1	CryoStor CS10	Before freezing	+	−
	CryoStor CS10	After freezing	+	−
	10% DMSO/90% FCS	Before freezing	−	−
	10% DMSO/90% FCS	After freezing	−	−
GBM #2	CryoStor CS10	Before freezing	+	+
	CryoStor CS10	After freezing	+	−
	10% DMSO/90% FCS	Before freezing	+	−
	10% DMSO/90% FCS	After freezing	+	−
GBM #3	CryoStor CS10	Before freezing	+	+
	CryoStor CS10	After freezing	−	−
	10% DMSO/90% FCS	Before freezing	+	−
	10% DMSO/90% FCS	After freezing	−	−
GBM #4	CryoStor CS10	Before freezing	+	−
	CryoStor CS10	After freezing	+	−
	10% DMSO/90% FCS	Before freezing	+	+
	10% DMSO/90% FCS	After freezing	+	−
GBM #5	CryoStor CS10	Before freezing	+	−
	CryoStor CS10	After freezing	+	+
	10% DMSO/90% FCS	Before freezing	+	+
	10% DMSO/90% FCS	After freezing	+	+
GBM #6	CryoStor CS10	Before freezing	+	−
	CryoStor CS10	After freezing	+	+
	10% DMSO/90% FCS	Before freezing	−	−
	10% DMSO/90% FCS	After freezing	+	+
GBM #7	CryoStor CS10	Before freezing	+	+
	CryoStor CS10	After freezing	+	+
	10% DMSO/90% FCS	Before freezing	+	−
	10% DMSO/90% FCS	After freezing	+	+
GBM #8	CryoStor CS10	Before freezing	+	−
	CryoStor CS10	After freezing	+	+
	10% DMSO/90% FCS	Before freezing	−	−
	10% DMSO/90% FCS	After freezing	−	−
GBM #1-8	CryoStor CS10	Before freezing	8+ of 8	3+ of 8
	CryoStor CS10	After freezing	7+ of 8	4+ of 8
	10% DMSO/90% FCS	Before freezing	5+ of 8	2+ of 8
	10% DMSO/90% FCS	After freezing	5+ of 8	3+ of 8

2D, two-dimensional; 3D, three-dimensional; DMSO, dimethyl sulfoxide; FCS, fetal calf serum.

**FIG. 3.**
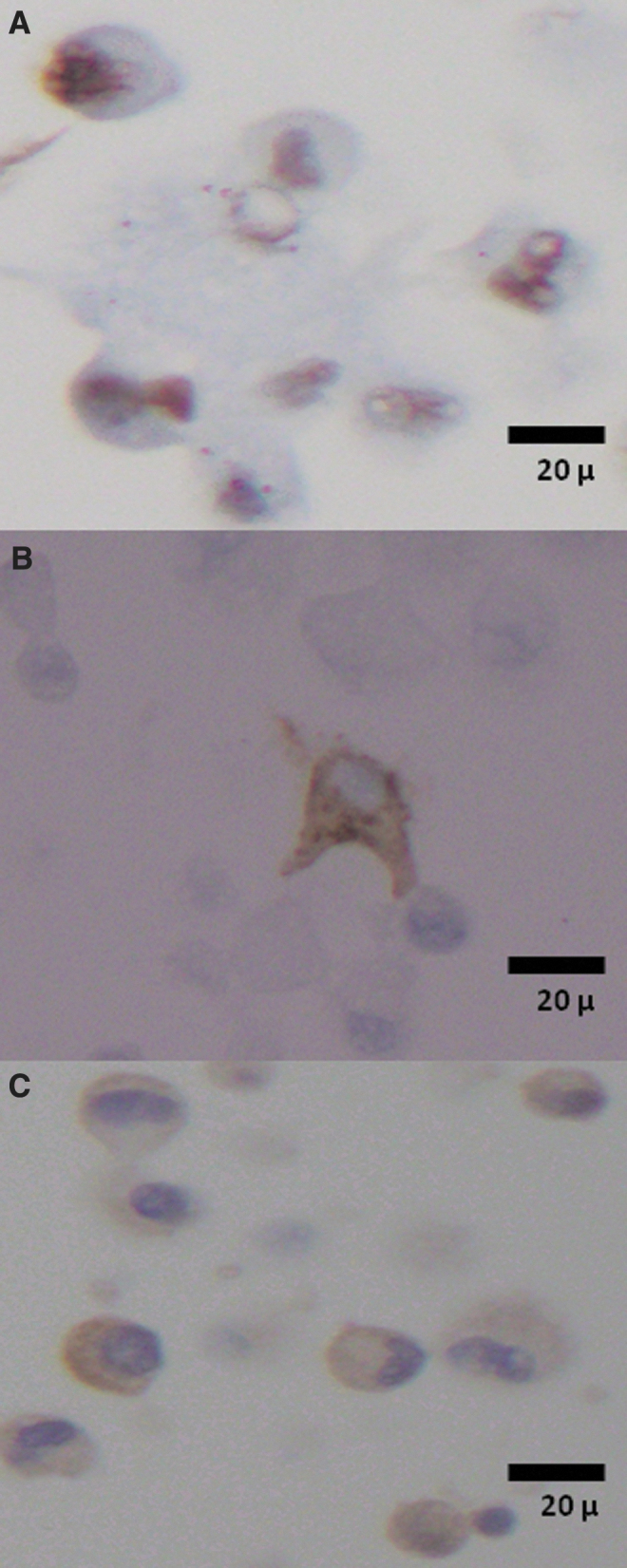
Nuclear expression of p53 **(A)** and cytoplasmic expression of GFAP **(B)** and the R132H mutant form of the IDH-1 protein **(C)** in cells of primary cultures derived from cryopreserved samples of GBM case #8.

Cryopreserved samples expressed GFAP, the R132H mutant form of the IDH-1 protein, or p53 in a pattern similar to that documented in the diagnostic pathology workup of the original tumor specimens (Table 3 and Fig. 3).

**Table 3.** Comparison of Immunohistochemical Features of Selected Primary Glioblastoma Cultures and Original Glioblastoma Specimens

Case no.	Immuno-stain	Original tumor specimens^a^	Cryopreservation^b^			
			Enzymatic digestion		Enzymatic digestion	
			Before cryopreservation		After cryopreservation	
			2D culture	3D culture	2D culture	3D culture
GBM #2	Mutant	0^c^	0	0	0	N/A
	IDH-1					
	P53	2+^c^	2+	2+	2+	N/A
	GFAP	1+^c^	1+	1+	1+	N/A
GBM #6	Mutant	0	0	N/A	0	0
	IDH-1					
	P53	1+	1+	N/A	1+	1+
	GFAP	1+	1+	N/A	1+	1+
GBM #8	Mutant	2+	2+	N/A	2+	2+
	IDH-1					
	P53	2+	2+	N/A	2+	2+
	GFAP	1+	1+	N/A	1+	1+

^a^ As documented for original diagnostic pathology specimen.

^b^ Tested primary GBM cultures originated from samples processed for cryopreservation in CryoStor CS10.

^c^ Results of immunohistochemical staining expressed in the following scale: 0 = no staining; 1+ = focal staining; 2+ = most cells staining.

NA, not applicable, no culture available for testing.

## Discussion

This study confirms the feasibility of viable glioblastoma cryopreservation and provides novel information about critical preanalytical determinants of the post-thaw viability of glioblastoma. We show here that no primary cultures could be produced from glioblastoma specimens that had been snap-frozen without the use of cryopreservation media. However, consistent with previous studies,^8^ we found that cryopreservation of viable glioblastoma is possible and allows for the establishment of primary glioblastoma cultures following thawing. We tested a series of cryopreservation protocols and found that the most efficient combination of upstream cryopreservation method and downstream culture conditions was the use of CryoStor CS10 cryopreservation medium, enzymatic digestion before freezing, and 2D post-thaw culturing, with a success rate of 8 out of 8 cases (100%). The immunohistochemical profiles of the established primary 2D or 3D cultures following cryopreservation were similar to the original surgical specimens. Our findings show that glioblastoma cryopreservation is fit for purpose, with the use of CryoStor CS10 cryopreservation medium after enzymatic digestion of fresh tissue, for establishment of primary tumor cultures.

Why CryoStor CS10 was better than 10% DMSO and 90% FCS is not evident from our experiments; however, several previous studies reported better cell survival following cryopreservation in a variety of experimental systems with the use of CryoStor solutions over a number of other cryopreservation media.^9–12^ CryoStor CS10 is a serum-free, protein-free, animal component-free intracellular-like defined cryopreservation medium containing 10% DMSO that has been designed to better maintain the ionic balance of cells at hypothermic and freezing temperatures and allow for more rapid recovery by reducing cryopreservation-induced stress and damage.^9–12^ The design of CryoStor CS10 includes pH buffering, free radical scavenging, oncotic/osmotic support, energy substrates, and ionic concentrations for increased balance at ultralow temperatures.^9–12^

We found that cryopreservation of glioblastoma tissue with enzymatic digestion before freezing was more effective for viable cryopreservation than cryopreservation without enzymatic digestion before freezing. Why enzymatic digestion before freezing was more effective is not evident from our experiments; however, previous studies reported both positive and negative effects of prefreeze enzymatic digestion on post-thaw cell viability using tissue types other than glioblastoma.^13,14^ Our findings together with previous observations by others suggest that enzymatic digestion before freezing may have a minor, tissue-specific positive or negative effect on tissue viability during cryopreservation.

Our study indicates that cryopreservation of mechanically minced glioblastoma tissue with enzymatic digestion before freezing was more effective for viable cryopreservation than cryopreservation of mechanically minced glioblastoma tissue without enzymatic digestion before freezing. However, it should be noted that there are limitations for enzymatic digestion before cryopreservation. These limitations primarily relate to the work- and time-intensive nature of enzymatic digestion that may not be easily performed routinely before cryopreservation. Cryopreservation of minced glioblastoma tissue in CryoStor CS10 without prior enzymatic digestion was nearly as efficient as the protocol with enzymatic digestion before cryopreservation.

While 3D tumor cell cultures can be very useful to study many aspects of tumor cell growth and therapy response,^15,16^ in our experiments, traditional 2D cultures provided a higher success rate than 3D cultures during post-thaw culturing of tumor cells. Although our study does not provide an explanation of this observation, possible causes may be related to growth inhibitory effects of the extracellular matrix in 3D cultures. Our experiments suggest that initial post-thaw culturing of glioblastoma cells should be performed in 2D.

Technologies related to the establishment of primary low passage glioblastoma cultures are increasingly important to neuro-oncology biobanking and research and a wide variety of protocols have been used and reported in the literature, without a clear consensus on optimal conditions.^4^ Importantly, to tissue biorepository practices, cryopreservation of viable glioblastoma specimens, allowing for primary culture months to years after surgical tumor collection and freezing, has been demonstrated.^8^ Our study points for the first time to some key determinants of the efficiency of viable glioblastoma cryobanking. We report an evidence-based optimized method, which can be fully validated in the future according to the requirements of ISO21899:2020 Biotechnology—Biobanking—General requirements for the validation and verification of processing methods for biological material in biobanks.^17^
